# Pathogenic correlation between mosaic variegated aneuploidy 1 (MVA1) and a novel *BUB1B* variant: a reappraisal of a severe syndrome

**DOI:** 10.1007/s10072-022-06247-w

**Published:** 2022-07-09

**Authors:** Piero Pavone, Xena Giada Pappalardo, Naira Mustafa, Raffaele Falsaperla, Simona Domenica Marino, Giovanni Corsello, Sebastiano Bianca, Enrico Parano, Martino Ruggieri

**Affiliations:** 1 Pediatric Clinic, Department of Clinical and Experimental Medicine, University Hospital A.U.O. “Policlinico-Vittorio Emanuele, Catania, Italy; 2 National Council of Research, Institute for Biomedical Research and Innovation (IRIB), Unit of Catania, Catania, Italy; 3 Department of Biomedical and Biotechnological Sciences (BIOMETEC), University of Catania, Catania, Italy; 4 Department of Paediatrics, School of Clinical Medicine, University of Cambridge, Cambridge, UK; 5 Department of Clinical and Chemical Pathology, Faculty of Medicine, Cairo University, Giza, Egypt; 6 Pediatrics and Pediatric Emergency Department, University Hospital, A.U.O “Policlinico Vittorio Emanuele”, Catania, Italy; 7 Mother and Child Department, Operative Unit of Pediatrics and Neonatal Intensive Therapy, University of Palermo, Palermo, Italy; 8 Medical Genetics, Referral Centre for Rare Genetic Diseases, ARNAS Garibaldi, Catania, Italy; 9 Unit of Rare Diseases of the Nervous System in Childhood, Department of Clinical and Experimental Medicine, Section of Pediatrics and Child Neuropsychiatry, University of Catania, AOU “Policlinico,” PO “G. Rodolico, Catania, Italy

**Keywords:** Mosaic variegated aneuploidy 1 (MVA1) syndrome, Microcephaly, Epileptic seizure, Ovary cyst, *BUB1B* gene

## Abstract

**Background:**

The BUB 1 mitotic checkpoint serine/threonine kinase B (BUB1B) gene encodes a key protein in the mitotic spindle checkpoint, which acts as a surveillance mechanism, crucial for the maintenance of the correct chromosome number during cell deviation. Mutations of *BUB1B* gene are linked to mosaic variegated aneuploidy 1 (MVA1) syndrome, a rare autosomal recessive disorder characterized by widespread mosaic aneuploidies, involving different chromosomes and tissues. MVA1 is clinically characterized by intrauterine growth restriction, post-natal growth retardation, and severe neurologic impairment including microcephaly, developmental delay/intellectual disability, epileptic seizures, and generalized hypotonia. Malignancies are also serious sequelae associated with the disorder. We reported on a case of two-year-old Italian girl with MVA1 who shows severe neurologic impairment, microcephaly and epileptic seizures.

**Materials and methods:**

Clinical data collection and genetic diagnosis of the patient were assessed. Mutational analysis covers the chromosomal microarray analysis, the gene methylation pattern studied using the methylation-specific multiplex ligation-dependent probe amplification, and the family-based Whole Exome Sequencing (WES). A literature research based on reported cases of MVA and premature chromatid separation was also included.

**Results:**

Karyotyping has revealed 12% of mosaics in the patient who carries a novel variant in *BUB1B* gene (c.2679A > T, p.Arg893Ser) detected by WES. Thirty-one cases of MVA1 including the present report, and four prenatally diagnosed cases with MVA1 were selected and inspected.

**Conclusion:**

Clinical and genetic findings reported in the girl strongly suggest a new MVA1 genotype–phenotype correlation and lead to a reappraisal of a severe syndrome. Diagnosis and in-depth follow-up provided worthwhile data.

**Supplementary Information:**

The online version contains supplementary material available at 10.1007/s10072-022-06247-w.

## Background

The *BUB1* mitotic checkpoint serine/threonine kinase beta (*BUB1B)* gene encodes for the protein BubR1 (budding uninhibited by benzimidazole-related-1). This protein has a central regulatory role of mitosis through phosphorylation of members of mitotic checkpoint complex (MCC), which subsequently bind to CDC20 protein preventing the activation of the anaphase promoting complex/cyclosome (APC/C), to ensure proper chromosome segregation in the metaphase before the progression to anaphase stage of mitosis [1]. The BubR1 protein is essential for the primary cilium formation and the characterization of mutations has been recently enclosed in the group of ciliopathies [2]. Mosaic variegated aneuploidy (MVA) is a group of rare autosomal recessive disorders characterized by premature chromatid separation (PCS) in > 50% metaphase cells and a variety of mosaic aneuploidies. MVA1 type 1 (OMIM 257,300) is caused by homozygous or compound heterozygous mutations in the *BUB1B* gene (OMIM 602,860) located in chromosome 15q1 and is clinically characterized by microcephaly, developmental delay/intellectual disability (DD/ID), epileptic seizures, generalized hypotonia, intrauterine growth restriction (IUGR), and postnatal growth retardation. Moreover, MVA1 may be associated with severe congenital malformations, and a high risk of malignancies such as rhabdomyosarcoma, Wilms tumor, myelodysplasia, and variable types of leukemia [3].

Herein, we report a case of 2-year-old girl who showed pre- and post-natal growth retardation, minor facial features, severe microcephaly with protruding metopic suture, epileptic seizures, generalized hypotonia, and congenital ovarian cyst. The genetic and genomic screening detected respectively in the patient 12% mosaic aneuploidies and a novel likely pathogenic variant (c.2679 A > T, p.Arg893Ser) of *BUB1B* gene. Clinical data were consistent with the diagnosis of MVA1 syndrome. A highly likely genotype/phenotype correlation between MVA1 and the novel *BUB1B* variant has been advanced.

## Patients’ medical report

**Fig. 1:**
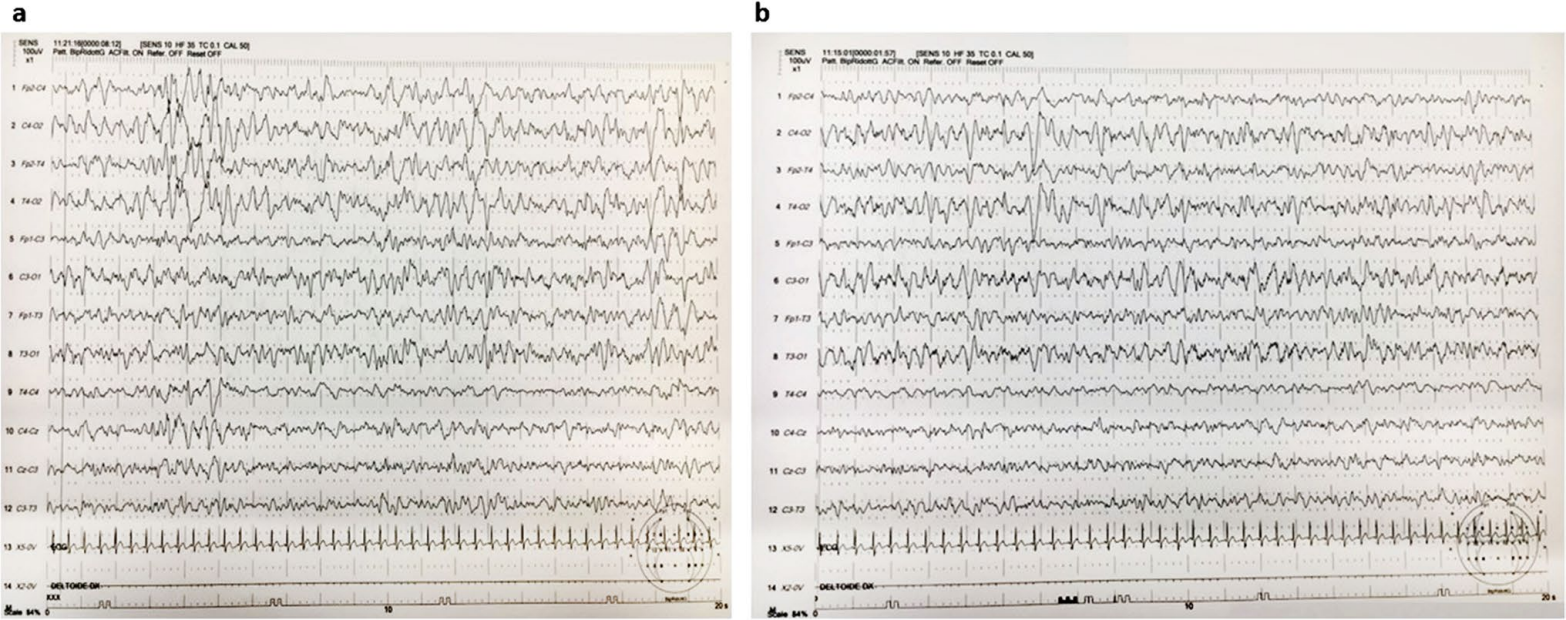
**a**, **b** EEG of the patient at the age of 2 years. EEG showing the dysregulated background and paroxysmal multifocal spike and waves more evident in the frontal regions

A 9-month-old female first came to the Department of Pediatrics, University Hospital Vittorio-Emanuele- Policlinico, Catania, Italy, for clinical checkup. Family history was irrelevant. The mother referred that at the age of 7 months of gestation, fetal ultrasound displayed the presence of a right ovarian cyst. Two weeks later, another ultrasound examination was done and revealed the presence of ascitic fluid with vanishing of the ovarian cyst. She reported normal fetal movements throughout the pregnancy. At the time of gestation, the ages of the little girl’s mother and father were 30 and 36 years old respectively. A programmed caesarean section was carried out with the birth of a female newborn with the following anthropometric measurements: weight 2190 g (3rd percentile), length 47 cm (50th percentile), and occipito-frontal circumference (OFC) 28 cm (< 3rd percentile). Apgar scores were 7 and 9 at 1 and 5 min respectively. The newborn was admitted to local Neonatal Intensive Care Unit (NICU) where she was oxygenated by intubation for four days. She was fed by nasogastric tube due to poor suckling reflex and refusal of alimentation. No signs of ascites were detected on a new abdominal ultrasound. At the age of 2 months and a half, she had an acute respiratory infection with severe respiratory distress, which required endotracheal intubation kept for 2 days. During the first months of life, she showed generalized hypotonia, feeding difficulty, and frequent episodes of vomiting. At 7th month, she had an epileptic seizure manifesting by fixed gaze and head rotation on the right side lasted few minutes and recovered by rectal midazolam. The EEG record was non-informative. Two days later, a new seizure came out with the same characteristic of the previous one and treatment with valproate was started. At 8th month, she was admitted to the Hospital of North Italy and discharged with a diagnosis of axial and segmental hypotonia, epileptic seizures, microcephaly with brain MRI of simplified cortical gyral pattern, and wide corpus callosum hypoplasia. At physical examination, the weight was 6800 g (3rd percentile), length 70 cm (50th), and OFC 38.5 cm (> 4th percentile). She showed minor facial features, microcephaly, developmental delay, generalized hypotonia, and growth retardation. The anterior fontanelle was open 0.5 × 0.5 cm and flat. Tendon reflexes were normally present. She was unable to hold her head up by arm traction maneuver. Routine laboratory analysis, electrolytes, plasma and urinary amino acid, organic acid, urinalysis, thyroid markers, sialo-transferrin, plasma purine, and total cholesterol were within normal limits. TORCH screen, inflammatory markers and otoacoustic emissions screening were normal. On abdominal ultrasound examination, no anomalies were found in the liver, spleen, kidneys, pancreas, or ovaries. ECG and echocardiogram were normal. EEG showed a background disorganization with the presence of rare spike and waves. Treatment with valproate and levetiracetam was continued. The little girl has been serially followed up in ambulatory care. During this period, she had three tonic clonic seizures of short duration, the last one was associated with fever. She presented recurrent stereotyped upper arm waving movements, raising and lowering upper limbs, beating the hands on the tables or other surfaces. The movement anomalies were seen during the day and with less frequency during sleep. At the current age of 2 years old, she has been newly admitted to the Institution. Her anthropometric measurements were weight 12 kg (25th–50th percentile), height 85 cm (50th percentile), and OFC 41.5 cm (< 4th percentile). At clinical examination, she showed minor facial features with notably small head and slightly protruding metopic suture, slanting forehead, short nose with round tip, right epicanthal fold, hypotelorism, down slanting palpebral fissures, small mouth, and dyschromic teeth. At the neurological examination, she showed generalized hypotonia, difficulty in holding her head up, and inability to sit up without support. The anterior fontanelle closed. Patellar reflexes were normally present. Routine laboratory analyses were normal. ECG, echocardiogram, fundus examination, and hearing exploration were also normal. Video-EEG at awake and during sleep showed paroxysmal multifocal spike and waves particularly evident in the bilateral frontal regions (Fig. 1a,b). No other seizures were recorded. Physiotherapy and antiepileptic treatment with valproate and levetiracetam were maintained.

## Results

**Fig. 2:**
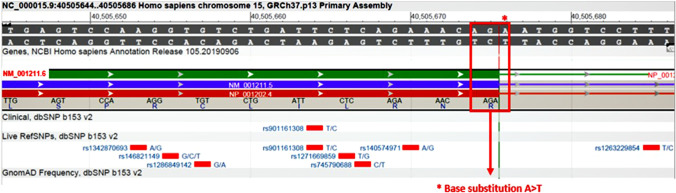
Graphical adaptation of the variant detection of *BUB1B* (NM_001211.6) gene from NCBI Variation Viewer (GRCh37/hg19). Highlighted red box shows the *BUB1B* variant (c.2679 A > T, p.Arg893Ser) detected in the patient

The results of CMA and MS-MLPA analysis were normal. Cytogenetic analysis revealed a normal karyotype in 88 cells (46, XX) and various aneuploidies in 12 cells. Among them, 7 cells had 45 chromosomes (45, XX,-10; 45, XX,-12; 45, XX,-18; 45, XX,-18; 45, X; 45, XX,-16; 45, XX,-5), only one had 44 chromosomes (44, XX,-14,-20). In two cells were observed a trisomy (47, XX, + 17; 47, XX, + 22), in another one was found a double trisomy (48, XX, + 1, + 16). A deletion of the long arm (q) of chromosome 10 was detected in a metaphase cell (46, XX,del10q). Trio WES analysis revealed no disease-causing variants, but a novel monoallelic (heterozygous) variant of uncertain significance (VUS) NM_001211.6: c.2679A > T, p.Arg893Ser in *BUB1B* gene was found in both the patient and her unaffected father. This variant has not been previously reported in the databases mentioned above (Fig. 2). By in silico analysis, the revealed VUS was predicted to be likely pathogenic affecting the catalytic function of the encoded protein.

## Discussion

The patient presented with pre- and post-natal growth retardation, severe primary microcephaly with pointed forehead, minor facial features, epileptic seizures partially responsive to treatment, generalized hypotonia, corpus callosum hypoplasia, and prenatal ovarian cyst. Clinical presentation associated with genetic findings revealing mosaic aneuploidies by karyotyping, and a novel heterozygous mutation (c.2679A > T, p.Arg893Ser) in *BUB1B* gene by WES analysis (Fig. 2) are consistent with a diagnosis of MVA1 syndrome. A likely correlation between clinical features presented by the patient the novel *BUB1B* variant seems to be highly probable. Subjects affected by MVA1 syndrome show a quite homogeneous and typical pattern of phenotypic manifestations as also observed in the present case. Kajii, Ikeuchi [3] reported five patients and reviewed other five cases from literature illustrating that all of these patients presented with similar clinical and cytogenetic findings, among which (a) more than 50% of mitotic lymphocytes with total PCS, (b) mosaic variegated aneuploidy in all the patients, (c) pre- and post-natal growth retardation, and profound developmental delay, (d) severe microcephaly, (e) hypoplasia of the brain with Dandy-Walker complex or other malformations of the posterior fossa, (f) drugs unresponsive clonic epileptic seizures, and (g) both parents with 3% or more mitotic lymphocytes in total PCS. As suggested by this study, less frequent features included minor facial features, bilateral cataracts, microphthalmia, cleft palate, ambiguous genitalia in males, skin abnormalities, and malignancies.

In Table 1 [4–23], we report clinical features of our patient and 30 MVA cases from the literature, while in Table 2, we list four known cases prenatally diagnosed with MVA1 with intrauterine fetal death or termination of pregnancy [24–27]. In the group of 30 MVA patients, pre-postnatal growth retardation, microcephaly, ID/DD, and epileptic seizures occurred in 93.5%, 87%, 77.4%, and 45.1% of the cases, respectively. Furthermore, DWM was present in 25.8% of the cases and other cerebral anomalies including the corpus callosum hypo-aplasia (CAA) were reported in 32.2% of the cases. Less frequent but serious presentations include genital anomalies (12.9%), renal dysfunction (12.9%), and endocrine dysfunction (12.9%). Malignancies had noticeably a high incidence (38.7%) with Wilms tumor, rhabdomyosarcoma and leukemia being the most frequently reported and recently polycystic ovary syndrome [21]. However, as mutations of *BUB1B* gene may cause microcephaly and other malformations in MVA1 subjects is a matter of debate. Simmons et al. 2019 [28] have demonstrated that BubR1 deficiency affects neural progenitors through impairing the mitotic checkpoint. Shortened mitosis, compromised genomic integrity, and massive cell death are considered the major causes of microcephaly in subjects with *BUB1B* mutations. Therefore, subjects who do not carry genetic mutations in *BUB1B* have milder phenotype with absence of microcephaly compared to those with BubR1 deficiency who show severe phenotype including microcephaly. According to these authors, nearly complete loss of BubR1 is also required for the pathogenesis of microcephaly [28].

**Table 1:** Systematic literature review of 31 MVA cases including the patient of present report

Author	Tolmie et al. 1988 [4]		Papi et al. 1989 [5]		Miller et al. 1990 [6]		Warburton et al. 1991 [7]		Nash et al. 1997 [8]	Flejter et al. 1998 [9]				Kajii et al. 1998 [10]		
Gender	F	M	M	F	M		F		F	F		F		F	M	
Pre/post–natal growth retardation	+	+	+	+	+		NA		+	+		+		+	+	
Microcephaly	+	+	+	+	+		+		+	+		+		+	+	
Facial dysmorphism	+	+	-	-	−		−		+	−		−		+	+	
DD/ID	+	+	+	+	+		+		−	−		−		+	+	
Epileptic seizures	+	−	−	−	+		−		−	−		−		+	+	
Other anomalies	Bilateral dislocated hip, naevus flammeus on the forehead, kyphoscoliosis, pectus carinatum	−	LGMD	LGMD	Immunodeficiency, ambiguous genitalia, hemangioma simplex on the forehead, thrombocytopenia		−		Pulmonary stenosis, glaucoma, hypothyroidism, clinodactyly	−		−		Bilateral cataract, HCH, DWM, cleft palate, multiple renal cysts	Bilateral cataract, DWM, HCH, ambiguous genitalia	
Malignancies	−	−	−	−	−		−		−	−		−		WT	−	
Author	Limwongse et al. 1999 [11]	Kawame et al. 1999 [12]	Plaja et al. 2001 [13]		Kajii et al. 2001 [14]							Lane et al. 2002 [15]		Jacquemont et al. 2002 [16]		Callier et al. 2005 [17]
Gender	M	M	F	F	M	F	F		M		M	M		M		M
Pre-postnatal growth retardation	NA	+	+	+	+	+	+		+		+	+		+		+
Microcephaly	+	+	+	+	+	+	+		+		+	+		+		NA
Facial dysmorphism	NA	+	−	−	−	−	+		+		+	+		+		+
DD/ID	+	+	+	+	+	+	+		+		+	+		+		+
Epileptic seizures	NA	+	−	−	+	+	+		+		+	+		−		−
Other anomalies	Cryptorchidis, clinodactyly	DWM, HCH	Hemangioma, thumb adduction	HCH, renal dysfunction	HCV, PFM	Bilateral cataract, HCH, CCa, DWM, HD	Bilateral cataract, microphthalmia, HCH, DWM		CCa, DWM, HCH, HD		CCa, DWM, HCH, multi-cystic lesions in bilateral kidneys	Short limb segments, epidermoid cysts, ventricular septal defect, subaortic stenosis, subnormal response to GH		−		Bilateral hip dislocation, hypospadias, curved penis, abducted thumbs, bilateral tapering, and bent second finger
Malignancies	RMS of the soft palate	WT	RMS	−	WT	WT	WT		RMS		WT	−		ALL		−
Author	Micale et al. 2007 [18]	Akasaka et al. 2013 [19]	Garcia-Castillo et al. 2008 [20]		Chaker et al. 2017 [21]		Kato et al. 2017 [22]	Ayaz et al. 2018 [23]			Present study		Total			%
Gender	M	M	M	F	F		M	F			F		14F/17 M			45.1
Pre-postnatal growth retardation	+	+	+	+	+		+	+			+		29			93.5
Microcephaly	NA	+	NA	+	−		+	+			+		27			87
Facial dysmorphism	+	−	+	+	+		+	+			+		19			61.2
DD/ID	+	+	−	−	−		−	+			+		24			77.4
Epileptic seizures	+	+	−	−	−		−	−			+		14			45.1
Other anomalies	Tapering fingers, small hands and feet, alternating exotropia hydronephrosis	DWM	Laryngeal cyst, hypothyroidism, café au lait spot, clinodactyly	Low thyroxin	Skeletal anomalies, knee varus, short fingers and toes		Bilateral cataract, ambiguous genitalia	Hearing loss Ankyloglossia Scoliosis Bilateral pes planus			CCa, prenatal ovary cyst		8 DWM			25.8
													10 other cerebral anomalies			32.2
													6 ocular anomalies			19.3
													4 endocrine dysfunctions			12.9
													4 genital anomalies			12.9
													4 renal dysfunctions			12.9
Malignancies	−	WT, Intraorbital tumor	−	−	Polycystic ovary		−	−			−		12			38.7

*ALL* acute lymphoblastic leukemia, *CCa* corpus callosum anomalies, *DD/ID* developmental delay/intellectual delay, *DWM* Dandy-Walker malformation, *GH* growth hormone, *HCH* hypoplasia cerebral hemisphere, *HCV* hypoplasia cerebellar vermis, *HD* hydrocephalus, *LGMD* limb girl muscular dystrophy, *NA* not available, *PFM* posterior fossa malformation;, *RMS* rabdomyosarcoma, *WT* Wils tumor, ( −) = absent;, ( +) = present

**Table 2:** MVA cases with intrauterine fetal death or termination of pregnancy

Author	Plaja et al. 2003 [24]	Chen et al. 2004 [25]	Cho et al. 2015 [26]	Yamaguchi et al. 2018 [27]
Gender	M	F	M	NR
Fetal malformation	Prenatal ultrasound shows a nuchal translucency	Microcephaly, facial dysmorphism, IUGR	Microcephaly, DWM	Growth restriction, microcephaly

*DWM* Dandy-Walker malformation, *IUGR* intrauterine growth restriction, *NR* not reported

**Fig. 3:**
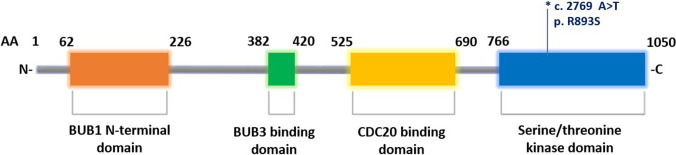
Schematic representation of BUB1B protein domain organization. In the protein structure can be identified an N-terminal region required for *BUB1B* binding, an intermediate region containing the BUB3 and CDC20 binding domains, and a catalytic serine/threonine kinase domain in the C-terminal region. The first three domains are involved in the interaction with the kinetochore, while the fourth is implicated in the regulation of the spindle assembly checkpoint (SAC). The symbol (*) indicates the position of the VUS detected in the present study. AA, amino acid

**Fig. 4:**
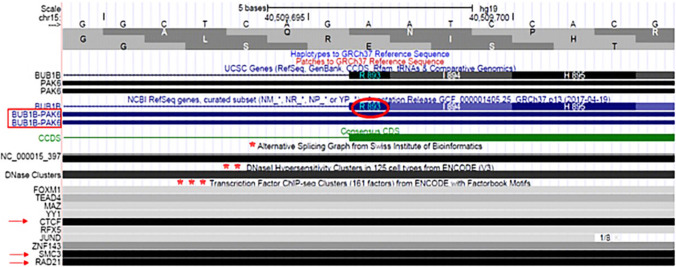
Gene regulation features of the missense variant c.2679A > T of *BUB1B* gene. UCSC Genome Browser (GRCh37/hg19) illustration of c.2679A > T of *BUB1B* gene. The amino acid substitution p.Arg893Ser (R893) of the missense variant c.2679A > T identified in the patient was highlighted in a red circle. The location of R893 is included in an active transcriptional region aligned with the sequence of the *BUB1B-PAK6* gene (shown on the left, in a red rectangle). The symbol (*) reports: the overlap of R893 with the large-scale annotations of alternative splicing variants indicating that the R893 is encoded across a splice junction; (**) the colocalization with DNase-I hypersensitive sites (DHSs), and (***) transcription factors binding sites (TFBS). The red arrows indicate the transcription binding factors for some proteins (CTCF, RAD21, and SMC3) implicated in chromatin remodeling

In the present study, the contribution of disease association for new low-penetrance *BUB1B* variant is determined. The revealed mutation in the patient and in her healthy father was predicted to be likely deleterious affecting the highly conserved catalytic domain of serine/threonine kinase subunit that may cause the loss of *BUB1B* kinase activity involved in the regulation of the signaling cascade of the spindle assembly checkpoint (SAC) components [29, 30], and other chromatin substrates, such as histones [31, 32] (Fig. 3). In addition, the variant seems to overlap with an active promoter region encoding for the readthrough transcripts of *BUB1B*-PAK6 gene, which product is the same as the downstream gene (PAK6) and was considered as a candidate gene for epileptic encephalopathy [4]. This can explain the epileptic seizures found in the patient. Furthermore, the overall framework emerged from ChIP-seq data (Fig. 4) indicates that the detected variant includes a conserved cluster of transcription factor binding site (TFBS) predicted to be targets for transcription factors implicated in the genomic stability. Therefore, the sequence variant may disrupt the gene expression and transcription regulatory motifs for CTCF, RAD21, and SMC3 proteins. Their co-distribution is suggestive to mediate the higher-order chromatin organization. The role of the insulator/repressor transcription factor CTCF and cohesins RAD21 and SMC3 has been investigated in the chromosomal instability (CI) leading to several tumors [5–7]. Recent studies on the clinical evaluation of VUS variants in some rare neurodevelopmental disorders have confirmed that the interpretation and the classification of VUS is influenced by the epigenetic signatures of the chromatin or changes in DNA methylation profile. Thus, the different methylation patterns of the entire affected gene may assist in the discrimination between a benign and pathogenic VUS in the same carriers [5, 6]. Considering the mosaic aneuploidies found in a small subset of cells, although the cytogenetic investigation was performed only in white blood cells and not repeated, aberrant chromosomal disjunctions of the reported case are representative of the causal mechanism leading to a defective cell division typically associated to mutations in *BUB1B* gene. A genotype–phenotype correlation can be assumed, despite cannot be fully proven for the limits of the heterozygosity, is compatible with the observed clinical framework that is mostly coincidental and overlapped with other known cases of literature affected by *BUB1B* mutations.

## Conclusion

The new *BUB1B* variant (c.2679A > T; p.Arg893Ser) should be enclosed as a potential risk of MVA1. When MVA diagnosis has been established after carrying out all relevant radiological and laboratory investigations, the management plan should be posed for the patients for early detection of malignancies. In infants or very young children, we suggest alerting parents about the nature of such disorder, and to keep in touch with the physician when some new clues arise. Abdominal ultrasound, fundus examination, and hematological checkup are advised every 3 months. Pathogenic mechanisms underlying MVA syndrome and the progression of related complications remain unclear. Up to now, one of the best described molecular events that can be attributed to MVA1 pathogenesis is the failure of mitotic checkpoint and ciliary functions caused by *BUB1B* mutations, which are indeed associated with a more severe disease phenotype and increased cancer risks. Therefore, follow-up of the patients and screening for malignancy is mandatory.

## Methods

### Data collection process

According to Preferred Reporting Items for Systematic Reviews and Meta-Analyses (*PRISMA*) statement, publications of reported cases of mosaic variegated aneuploidy 1 (MVA1) syndrome and premature chromatid separation (PCS) were retrieved through PubMed, Cochrane Library, and Scopus Web of Science database. A literature research used in this review provided 30 MVA1 cases, and 4 prenatally diagnosed with MVA1 with intrauterine fetal death or termination of pregnancy. From the selected studies all relevant and available clinical data were extracted. Data items such as number and gender of patients, pre-postnatal growth retardation, microcephaly, facial features, DD/ID, epileptic seizures, other anomalies, malignancies and fetal malformations were reported in Tables 1 and 2.

### Genetic testing and data analysis

A karyotyping on 100 metaphases from peripheral blood mononuclear cells of the patient was performed. Using blood-derived genomic DNA, chromosomal microarray analysis (CMA) was conducted using the CytoSNP‐850 K array (Illumina) v.1. Data analysis was done using Bluefuse Multisoftware v.4.4 (Bluegnome, UK) according to the manufacturer’s instructions. To determine additional chromosome abnormalities and the methylation status associated with the 15q11-13 region, methylation-specific multiplex ligation-dependent probe amplification (MS-MLPA) was performed. The trio whole exome sequencing (trio WES) analysis was employed in the patient and her parents using the NimbleGen SeqCap EZ kit (Roche) for target enrichment and sequencing on the Illumina MiSeq platform following the manufacturer’s protocol. Sanger sequencing was then used to validate WES results. NGS data analysis and an in-house filtering process were done using Isis (Analysis Software) 2.5.1.3; BWA (Aligner) 0.6.1-r104-tpx; SAM tools 0.1.18 (r982295) and GATK (Variant Caller). The clinical interpretation of genomic variant was done by ClinVar, Human Gene Mutation Database (HGMD), Leiden Open Variation Database (LOVD, v.3.0), and Genome Aggregation Database (gnomAD, v.3). Variants were classified as pathogenic/likely pathogenic/VUS/likely benign/benign, according to the 2015 American College of Medical Genetics and Genomics (ACMG) guidelines [33]. Prediction of pathogenicity of non-synonymous variants was determined by in silico tools such as Sift, Provean, MutationTaster, and Regulation Spotter.

## Supplementary Information

Below is the link to the electronic supplementary material.

Supplementary file1 (DOCX 14 kb) **S1. In-silico mutation analysis for the query chr15:40505676 A>T was performed to evaluate DNA sequence variants for their disease-causing potential. **Results were reported below by the following free web-based tools: SIFT (https://sift.bii.a-star.edu.sg/); Provean (http://provean.jcvi.org/genome_submit_2.php?species=human); MutationTaster (https://www.genecascade.org/MutationTaster2021/#transcript); RegulationSpotter (https://www.regulationspotter.org/RegulationSpotter/AnalyseVariant.html).

## Data Availability

All data generated or analyzed during this study are included in this published article and may be released upon application to the corresponding author who can be contacted at ppavone@unict.it.

## Abbreviations

*BUB1B*: BUB1 mitotic checkpoint serine/threonine kinase beta
CMA: Chromosomal microarray analysis
DD/ID: Developmental delay/intellectual disability
IUGR: Intrauterine growth restriction
MVA: Mosaic variegated aneuploidy
PCS: Premature chromatid separation
VUS: Variant of uncertain significance
