# Supplementary material for: Pathogenic correlation between mosaic variegated aneuploidy 1 (MVA1) and a novel BUB1B variant: a reappraisal of a severe syndrome
Source: Neurol Sci. 2022 Jul 9;43(11):6529–38. doi: 10.1007/s10072-022-06247-w (PMC9616775; doi:10.1007/s10072-022-06247-w)
Supplement: Supplementary file 1 — Supplementary file1 (DOCX 14 kb) S1. In-silico mutation analysis for the query chr15:40505676 A>T was performed to evaluate DNA sequence variants for their disease-causing potential. Results were reported below by the following free web-based tools: SIFT (https://sift.bii.a-star.edu.sg/); Provean (http://provean.jcvi.org/genome_submit_2.php?species=human); MutationTaster (https://www.genecascade.org/MutationTaster2021/#transcript); RegulationSpotter (https://www.regulationspotter.org/RegulationSpotter/AnalyseVariant.html). [file 10072_2022_6247_MOESM1_ESM.docx]

**Table 2. In*-silico* mutation analysis for the query chr15:40505676 A>T was performed to evaluate DNA sequence variants for their disease-causing potential.**

| **Analyzed**  **features**  **Tools** | **Substitution** | **Region** | **Known variant^1^** | **SNP Type** | **Protein conservation** | **Splice effect** | **Prediction Score** |
| --- | --- | --- | --- | --- | --- | --- | --- |
| **SIFT**  (https://sift.bii.a-star.edu.sg/) | R893S | exon  CDS | novel | non-synonymous | highly phylogenetic conserved | splice site changes in exon-intron border and protein features might be affected | deleterious |
| **Provean** (http://provean.jcvi.org/genome_submit_2.php?species=human) |  |  |  |  |  |  | neutral |
| **MutationTaster** (https://www.genecascade.org/MutationTaster2021/#transcript) |  |  |  |  |  |  | damaging |
| **RegulationSpotter** (https://www.regulationspotter.org/RegulationSpotter/AnalyseVariant.html) |  |  |  |  |  |  | likely effect functional region |

Abbreviations: SNP (single nucleotide polymorphism); CDS (coding sequence).

^1^ Known variant in dbSNP, ExAC, 1000G, gnomAD.
